# Validation of the Spanish version of the migraine disability assessment questionnaire (MIDAS) in university students with migraine

**DOI:** 10.1186/s12883-020-01646-y

**Published:** 2020-02-24

**Authors:** Daniel Rodríguez-Almagro, Alexander Achalandabaso, Alma Rus, Esteban Obrero-Gaitán, Noelia Zagalaz-Anula, Rafael Lomas-Vega

**Affiliations:** 1 Department of Health Sciences, (Building B3, Office 205), University of Jaén, Paraje, Campus Las Lagunillas s/n, 23071 Jaén, Spain; 2 Department of Cell Biology, University of Granada, Avenida de Fuente Nueva s/n, 18071 Granada, Spain

**Keywords:** Headache, Migraine disorders, Disability evaluation, Reproducibility of results, Surveys and questionnaires

## Abstract

**Background:**

The Migraine Disability Assessment (MIDAS) questionnaire is widely used to determine the degree of migraine-related disability of subjects. So far, and to the best of our knowledge, no Spanish version of this tool has been validated. The questionnaire comprises seven items, with the first five constituting the main scale while the sixth and seventh items referring, respectively, to the frequency and intensity of headache. The present study aims to analyze the clinimetric properties of the Spanish version of the MIDAS questionnaire in a population of university students.

**Methods:**

We performed a cross-sectional study of validation for this measuring instrument. A total of 153 subjects participated in the study. We analyzed construct validity using factor analysis, test-retest reliability by the Intraclass Correlation Coeficient (ICC), internal consistency, and concurrent validity with respect to the 12-Item Short Form Health Survey (SF-12).

**Results:**

Factor analysis revealed a two-factor structure. The questionnaire has good reliability for the MIDAS main-scale score ([ICC = 0.81; 95% CI: 0.63–0.90]), excellent reliability for headache frequency (ICC = 0.90; 95%; CI: [0.79–0.95]), and moderately good reliability for headache intensity (ICC = 0.63; 95% CI: [0.34–0.80]). The analysis also yielded good internal consistency results (α Cronbach = 0.797) and a moderate correlation between MIDAS-main scale and the physical component summary of SF-12 (Rho = − 0.326; *p* <  0.001).

**Conclusions:**

The Spanish version of the MIDAS questionnaire is a valid and reliable tool to measure migraine-related disability in university subjects. The two additional items provide information that could help clinicians in making decisions.

## Background

Migraine is one of eight disorders that affect over 10% of the world population [1]. It is one of the most common chronic pain conditions among the young adult population [2], with prevalence peaking between 20 and 30 years of age. Women [3] and students are some of the populations most widely affected by migraine [4]. With a large prevalence of around 37%, it involves a mean annual cost per person of €1222, with Spain having the highest total cost in Europe [3]. Since 1990 the number of years lived with disability caused by migraine has increased by 51.2% [5].

The Migraine Disability Assessment (MIDAS) questionnaire was developed as a tool to determine headache-related disability [6]. It estimates productive time lost to the disabling effects of headache over the three preceding months.

MIDAS has been transculturally adaptated to numerous languages, and has been widely used for research purposes and in clinical practice to develop treatment strategies based on the patient’s level of disability [7]. To the best of our knowledge, there is no validated Spanish version of the MIDAS questionnaire, and its clinimetric properties in that context remain unknown. However, several studies have used the MIDAS questionnaire in Spanish populations to determine both migraine-related disability [8, 9] and the difficulty encountered by patients when using the questionnaire [10].

Given the high prevalence and costs of migraine, particularly in Spain, and the lack of a reliable tool, written in Spanish, to measure migraine-related disability, we have analyzed the clinimetric properties of the Spanish version of the MIDAS questionnaire in university students, one of the populations most commonly affected by migraine.

## Material and methods

### Design

This is an observational, cross-sectional study for the validation of a measuring instrument, in which we have analyzed the construct validity, internal consistency, and concurrent validity of the MIDAS questionnaire. For test-retest reliability, a subsample of 29 subjects was evaluated twice with a time interval of 21 days.

### Participants

The participants were young adult undergraduate and graduate students over 18 years. In order to be included, all participants were required to have been diagnosed with migraine. They were then examined by a physician (F.H.) to check if they fulfilled at that time the criteria described in the third edition of *The International Classification of Headache Disorders* [11].

**Fig. 1:**
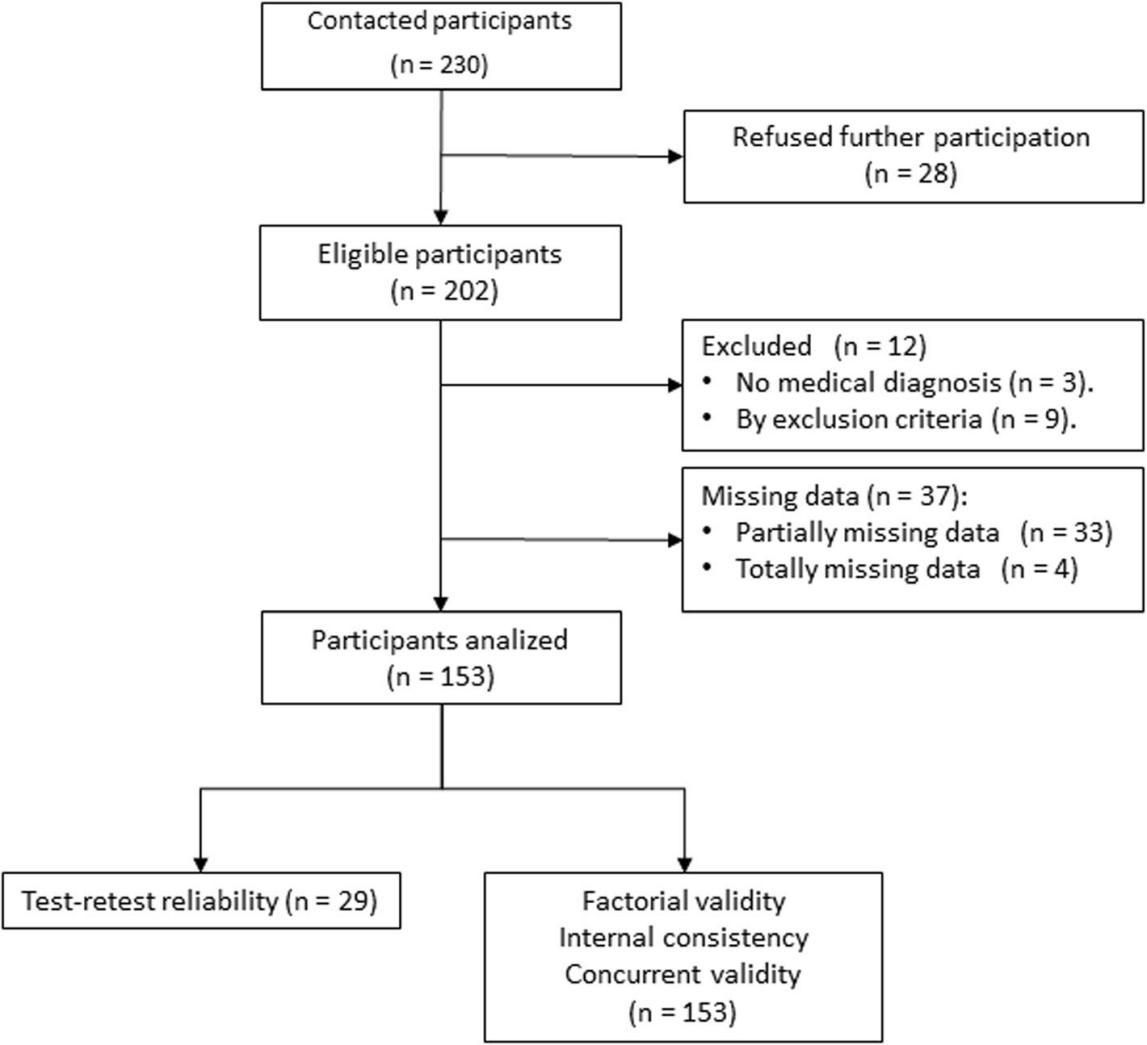
Flow diagram of participants

We followed recommendations to include at least 10 subjects per item and to have at least 100 subjects for the purposes of the internal consistency and factor analysis [12]. A total of 230 subjects were contacted within the University of Jaén (Spain), of which 202, aged between 18 and 33, participated in this study between the months of March and June 2017. Finally, 153 participants met the inclusion criteria and completed all tasks. The selection process is shown in Fig. 1.

The present study was approved by the Bioethics Committee of the University of Jaén (Reference number ABR 7/17) and was developed in accordance with the Helsinki Declaration, good clinical practices, and all applicable laws and regulations. All participants provided written informed consent prior to their inclusion in the study.

### Measurements

Prior to completing the questionnaires, the participants reported their sociodemographic data including age, gender, weight, height, smoking habit, and degree of physical activity.

Two bilingual experts performed independent translations of the English version of the MIDAS questionnaire [6] into Spanish, following the guidelines recommended by the International Quality of Life Assessment project for cross-cultural translation [13]. Secondly, a consensus for a preliminary forward translation was reached between translators and researchers. Later, two bilingual experts performed a backward translation of the agreed Spanish version into English. The last English translation was compared with the original version of the MIDAS questionnaire in order to verify whether they had achieved semantic, linguistic, conceptual, and technical equivalence. Finally, 15 participants filled the Spanish version of the questionnaire (Additional file 1) to verify if the instructions, questions, and answering options were understandable.

The MIDAS questionnaire measures the degree of migraine-related disability experienced over the last 3 months. It comprises seven items, the first five of which constitute the main scale and inquire about three distinct dimensions: workplace (two first items); domestic tasks (third and fourth items); and attendance to social, family, or leisure activities (fifth item). The sixth and seventh items refer respectively to the frequency and intensity of headaches over the last 3 months, and provide relevant data for clinicians to make informed decisions. The first six items must be answered with the number of days that headache conditioned any of the activities described in each question over the last 3 months. The seventh item is a Numeric Pain Scale, in which zero indicates absence of pain and ten indicates the maximum pain subjects believe they can withstand. According to the MIDAS-main scale score, which comprises the sum of the answers to items 1 to 5, subjects could be classified in 5 disability grades: Grade I (score 0–5): no disability or low disability; Grade II (score 6–10): mild disability; Grade III (score 10–20): moderate disability; and Grade IV (score > 21): severe disability.

The 12-Item Short Form Health Survey (SF-12) was used in the present study to measure quality of life [14]. This is a self-administered questionnaire extracted from the SF-36 by means of multiple regression. The SF-12 consists of 12 items from which the physical and mental component summaries (PCS-12 and MCS-12, respectively) yield a single score each. These two summary components showed high levels of internal consistency (Cronbach’s alpha value of 0.85 for PCS-12 and 0.78 for MCS-12) [14].

### Statistical analysis

Data management and analysis were performed using the IBM SPSS Statistics package, version 23.0 (SPSS Inc., Chicago, IL) and the MedCalc statistical software, version 16.5.4 (MedCalc Software bv, Ostend, Belgium; https://www.medcalc.org; 2019). Data were expressed as means and standard deviations (SD) for continuous variables and as frequencies and percentages for categorical variables. The Kolmogorov-Smirnov test was used to test the normality of continuous variables. The level of statistical significance was set at *P* <  0.05.

A Principal Component Analysis (PCA) with varimax rotation was performed to measure the validity of the MIDAS construct. To test the feasibility of the factorial analysis we used Barlett’s sphericity test. The suitability of the sample was analyzed using the Kaiser-Meyer-Olkin (KMO) test.

Internal consistency of the instrument was assessed through item analysis and the calculation of Cronbach’s alpha. Values of Cronbach’s alpha below 0.70 were considered weak, between 0.70 and 0.90 were considered good, and above 0.90 were interpreted as indicative of item redundancy [15].

Test-retest reliability was analyzed using the Intraclass Correlation Coeficient (ICC) as described by Shrout & Fleiss. Reliability was considered low for ICC values below 0.40, moderate for values between 0.40 and 0.75, high for ICC values between 0.75 and 0.90, and excellent for values higher than 0.90 [16].

Spearman’s correlation coefficient was used to analyze the tool’s concurrent validity with the SF-12 questionnaire. A correlation coefficient greater than 0.5 indicated a strong correlation, whereas values between 0.30 and 0.50 indicated a moderate correlation [17].

## Results

Out of the 153 subjects who completed the study (average age = 21.76; SD = 3.65), 45 were men and 108 were women. According to the disability level, 41.8% of participants showed little to no disability, 32% showed mild disability, 16.3% showed moderate disability, and 9.8% showed severe disability (Table 1).

**Table 1:** Description of the participants

VARIABLES		Migraineurs (*n* = 153)	
CONTINUOUS		Mean	SD
Age		21.76	3.65
Height (cm)		168.18	7.95
Weight (kg)		64.93	11.95
BMI (kg/m^2^)		22.85	3.17
PCS-12		52.42	7.45
MCS-12		41.93	11.49
Frequency of headache (Item 6)		10.11	14.84
Pain intensity (Item 7)		5.48	2
CATEGORICAL		F	%
Gender	Male	45	70.6
	Female	108	29.4
Smoker	Yes	21	13.7
	No	132	86.3
Physical activity	Yes	66	43.1
	No	87	56.9
Kind of migraine	Migraine	120	78.4
	Chronic migraine	33	21.6
Attack duration	4 h	73	47.7
	4-24 h	62	40.5
	> 24	18	11.8
Photophobia	Yes	84	54.9
	No	69	45.1
Phonophobia	Yes	104	68
	No	49	32
Nausea or Vomiting	Yes	90	58.8
	No	63	41.2
Disability grade (MIDAS)	No disability	64	41.8
	Mild	49	32
	Moderate	25	16.3
	Severe	15	9.8

*BMI* Body Mass Index, *PCS-12* Physical Component Summary of the 12-Item Short Form Health Survey (SF-12), *MCS-12* Mental Component Summary of the SF-12, *MIDAS* Migraine Disability Assessment

The PCA showed a structure composed of two factors. The first factor included items 1, 3, and 5, which are questions that imply refraining from engaging in activities, regardless of type, due to headache. The second factor included items 2 and 4, which imply a decrease of 50% in the performance at work or at domestic tasks (Table 2). The variance explained was 88.35%, Bartlett’s sphericity test was statistically significant (X^2^ = 707.97; *p* <  0.001), and the KMO index was 0.612, indicating that the sample used can be considered adequate for the factor analysis.

**Table 2:** Percentages of variance explained by the factor analysis performed using Principal Components Analysis

Component	Initial eigenvalues			Extraction sums of squared loadings			Rotation sums of squared loadings		
	Total	% of variance^a^	Cumulative %^b^	Total	% of variance^a^	Cumulative %^b^	Total	% of variance^a^	Cumulative %^b^
1	2.846	56.922	56.922	2.846	56.922	56.922	2.823	56.460	56.460
2	1.571	31.428	88.350	1.571	31.428	88.350	1.595	31.890	88.350
3	0.429	8.585	96.935						
4	0.113	2.254	99.188						
5	0.041	0.812	100.000						

^a^Percentage of variance that explains each factor of the questionnaire structure

^b^Total percentage of variance explained jointly by the factors that compose the questionnaire structure

Internal consistency analysis showed a Cronbach’s alpha of 0.797 for the MIDAS-main scale score, which may improve slightly if items 2 and 4 were eliminated (Table 3). In addition, test-retest reliability was high for the MIDAS-main scale score (ICC = 0.81 95% CI: 0.63–0.90 *p* <  0.001), excellent for item 6 (headache frequency; ICC = 0.90 95%; CI: 0.79–0.95; *p* <  0.001), and moderate for item 7 (headache intensity; ICC = 0.63; 95% CI: 0.34–0.80; *p* <  0.001). The analysis showed a strong correlation of the MIDAS-main scale score with headache frequency, and a moderate correlation with both headache intensity and PCS-12. Additionally, moderate correlations were found between PCS-12 scores and the frequency and intensity of headaches (Table 4).

**Table 3:** Item reliability analysis

	Average scale if the item is deleted	Variance of scale if the item is deleted	Corrected items - total correlation	Cronbach’s alpha if the item is deleted^a^
Item 1	10.654	302.333	0.832	0.661
Item 2	8.765	538.878	0.134	0.851
Item 3	9.078	288.625	0.846	0.653
Item 4	9.137	523.040	0.203	0.842
Item 5	9.686	287.677	0.878	0.639

Item 1–5: questions in the MIDAS questionnaire

^a^Cronbach’s alpha value if the item is deleted from the analysis

**Table 4:** Correlations among MIDAS-main scale score. Frequency and intensity of headache, and physical and mental components summary of the SF-12 questionnaire

	MIDAS-main scale score		Headache frequency		Headache intensity	
	Rho Spermann	*P*	Rho Spermann	*P*	Rho Spermann	*P*
Headache frequency	0.529	< 0.001				
Headache intensity	0.343	< 0.001	0.459	< 0.001		
PCS-12	−0.326	< 0.001	−0.334	< 0.001	−0.268	< 0.001
MCS-12	−0.153	0.060	−0.029	0.721	−0.027	0.741

*MIDAS* Migraine Disability Assessment, *PCS-12* Physical Component Summary of the 12-Item Short Form Health Survey (SF-12), *MCS-12* Mental Component Summary of the SF-12, *MIDAS* Migraine Disability Assessment

## Discussion

The MIDAS questionnaire has been used in Spanish populations to assess migraine-related disability [8–10]. However, to the extent of our knowledge, there is no validated Spanish version of the questionnaire, and this is the first study to analyze the clinimetric properties of the Spanish version of the MIDAS questionnaire. Our results for a population of university students show a factorial structure composed of two factors, good internal consistency data, results ranging from good to excellent in test-retest reliability, and a moderate correlation with PCS-12 in the concurrent validity analysis of the questionnaire. Therefore, our results indicate that the Spanish version of the MIDAS questionnaire is a valid and reliable instrument to measure migraine-related disability.

However, our results showed a different factorial structure to the one proposed by the original authors [6]. Unlike the original structure, our PCA clearly identified two factors that explained nearly 90% of the variance. The first factor was composed of items that imply refraining from certain activities, such as working, doing house chores, or attending social events, due to migraine (items 1, 3, and 5). The second factor was composed by items that involve a 50% decrease in performance of both work-related and domestic tasks because of migraine (items 2 and 4). This two-factor structure is indicative of the variable influence that migraine has on personal performance and, consequently, on disability.

In our population, nearly two thirds of migrainous women and men presented some kind of disability, and this was related to decreased physical quality of life. These results are in agreement with previous studies [18, 19]. In addition to its physical effects, several cognitive functions such as processing speed, attention, memory, verbal skills, and executive function, which are all particularly important for the daily performance of our population, are negatively affected by migraine [20] and their debilitating consequences have a considerable impact on daily-life activities [21]. In the present study, the first factor of the factorial structure includes all items that reflect total disability for the purposes of engaging in any professional or social activity, while the second factor reflects a partial disability level.

The reliability parameters of the Spanish version of the MIDAS questionnaire were satisfactory as a whole. We have obtained good internal consistency results, comparable to those previous studies [18, 19, 22–24] including the most recent ones [25, 26]. Results from the test-retest reliability analysis ranged from good to excellent. Our analysis also revealed that the two additional items of the questionnaire are reliable and provide relevant information that may be helpful for clinicians, in agreement with statements made by the original authors of the questionnaire [6]. The results obtained are in accordance with the procedure for the measurement of migraine-related disability proposed by the original authors, although the factorial structure shown in the present study was different. In fact, it suggests that migraine-related disability may be assessed differently, given the specific effects of migraine on cognitive functions regardless of its severity, thereby generating different disability levels that may lead from absenteeism to presentism [4].

The results of the present study show good clinimetric properties for the Spanish version of the MIDAS questionnaire, evidencing that this version is a consistent and reliable measure tool.

This study has several limitations. Although the prevalence of migraine is high among student populations, our results are only valid for the sample under analysis and not be extrapolated to other populations. In addition, our results may only be valid for a Spanish population due to structural and organizational differences among the educational systems of different countries.

Future studies should analyze the clinimetric properties of the MIDAS questionnaire for different populations and countries. It would be also advisable to examine the relation between headache and other concomitant disorders such as neck pain or dizziness, as well as to analyze the factors related to the presence of headache in university students and the impact that this disorder may have on this population.

## Conclusions

The Spanish version of the MIDAS questionnaire is a valid and reliable tool for measuring migraine-related disability in young university students. Moreover, the two additional items of the questionnaire provide information that can help clinicians discriminate between subjects with and without headache, and enable a wider application of this scale.

## Supplementary information

**Additional file 1.** Spanish version of the Migraine Disability Assessment (MIDAS) questionnaire.

## Data Availability

The datasets used and/or analysed during the current study are available from the corresponding author on reasonable request.

## Abbreviations

ICC: Intraclass Correlation Coeficient
KMO: Kaiser-Meyer-Olkin test
MCS-12: Mental Component Summary
MIDAS: Migraine Disability Assessment
PCA: Principal Component Analysis
PCS-12: Physical Component Summary
SD: Standard Deviation
SF-12: 12-Item Short Form Health Survey

## References

[1] Global Burden of Disease Study 2013 Collaborators (2015). Global, regional, and national incidence, prevalence, and years lived with disability for 301 acute and chronic diseases and injuries in 188 countries, 1990–2013: a systematic analysis for the Global Burden of Disease Study 201. Lancet.

[2] You DS, Albu S, Lisenbardt H, Meagher MW (2019). Cumulative childhood adversity as a risk factor for common chronic pain conditions in young adults. Pain Med.

[3] Steiner TJ, Stovner LJ, Katsarava Z (2014). The impact of headache in Europe: principal results of the Eurolight project. J Headache Pain.

[4] Woldeamanuel YW, Cowan RP (2017). Migraine affects 1 in 10 people worldwide featuring recent rise: a systematic review and meta-analysis of community-based studies involving 6 million participants. J Neurol Sci.

[5] GBD 2016 Headache Collaborators (2018). Global, regional, and national burden of migraine and tension-type headache, 1990–2016: a systematic analysis for the Global Burden of Disease Study. 2016. Lancet Neurol.

[6] Stewart WF, Lipton RB, Whyte J (1999). An international study to assess reliability of the migraine disability assessment (MIDAS) score. Neurology..

[7] El Hasnaoui A, Doble A, Gaudin AF (2006). Tools for assessing patient perception of the impact of migraine. CNS Drugs.

[8] Garcia ML, Baos V, Lainez M, Pascual J, Lopez-Gil A (2008). Responsiveness of migraine-ACT and MIDAS questionnaires for assessing migraine therapy. Headache.

[9] González-Quintanilla V, Toriello-Suárez M, Gutiérrez-González S (2015). Stress at work in migraine patients: differences in attack frequency. Neurologia..

[10] Medrano Martínez V, Francés Pont I, Hernández Rubio L, González Fernández L, Fernández Izquierdo S, Mallada Frechin J. Perception of the validity of the migraine disability assessment questionnaire in a population of patients with chronic migraine. Neurologia. 2018;pii: S0213–4853(18)30195–6. 10.1016/j.nrleng.2020.08.004 34752347

[11] Headache Classification Committee of the International Headache Society (IHS) (2013). The International Classification of Headache Disorders, 3rd edition (beta version). Cephalalgia.

[12] Kline P (1993). An easy guide to factor analysis.

[13] Bullinger M, Alonso J, Apolone G (1998). Translating health status questionnaires and evaluating their quality: the IQOLA Project approach. International quality of life assessment. J Clin Epidemiol.

[14] Vilagut G, Valderas JM, Ferrer M (2008). Interpretation of SF-36 and SF-12 questionnaires in Spain: physical and mental components. Med Clin (Barc).

[15] Tavakol M, Dennick R (2011). Making sense of Cronbach’s alpha. Int J Med Educ.

[16] Shrout PE, Fleiss JL (1979). Intraclass correlations: uses in assessing rater reliability. Psychol Bull.

[17] Cohen J (1988). Statistical analysis for the behavioral sciences.

[18] Georgoudis G, Parasxou A, Chara P (2015). Functional assessment in Greek tension-type headache sufferers: validity, reliability, responsiveness and psychometrics of the migraine disability assessment questionnaire (MIDAS). Br J Med Med Res.

[19] Zandifar A, Asgari F, Haghdoost F (2014). Reliability and validity of the migraine disability assessment scale among migraine and tension type headache in Iranian patients. Biomed Res Int.

[20] Vuralli D, Ayata C, Bolay H (2018). Cognitive dysfunction and migraine. J Headache Pain.

[21] Lindbergh CA, Dishman RK, Miller LS (2016). Functional disability in mild cognitive impairment: a systematic review and meta-analysis. Neuropsychol Rev.

[22] Shaik MM, Hassan NB, Tan HL, Bhaskar S, Gan SH (2014). Validity and reliability of the Bahasa Melayu version of the migraine disability assessment questionnaire. Biomed Res Int.

[23] Juyal R, Verma R, Garg RK, Shukla R, Agarwal A, Singh MK (2010). Reliability and validity of Hindi translation of the migraine disability assessment and headache impact test-6 questionnaires. Ann Indian Acad Neurol.

[24] D'Amico D, Grazzi L, Usai S (2003). Use of the migraine disability assessment questionnaire in children and adolescents with headache: an Italian pilot study. Headache..

[25] Oikonomidi T, Vikelis M, Artemiadis A, Chrousos GP, Darviri C (2018). Reliability and validity of the Greek migraine disability assessment (MIDAS) questionnaire. Pharmacoecon Open.

[26] Benz T, Lehmann S, Gantenbein AR (2018). Translation, cross-cultural adaptation and reliability of the German version of the migraine disability assessment (MIDAS) questionnaire. Health Qual Life Outcomes.

