# Supplementary material for: Validation of the Spanish version of the migraine disability assessment questionnaire (MIDAS) in university students with migraine
Source: BMC Neurol. 2020 Feb 24;20:67. doi: 10.1186/s12883-020-01646-y (PMC7038557; doi:10.1186/s12883-020-01646-y)
Supplement: Supplementary file 1 — Additional file 1. Spanish version of the Migraine Disability Assessment (MIDAS) questionnaire. [file 12883_2020_1646_MOESM1_ESM.docx]

**Cuestionario MIDAS**

INSTRUCCIONES: Por favor, responda a las siguientes preguntas respecto a TODOS los dolores de cabeza que haya tenido durante los últimos tres meses. Escriba su respuesta en las celdas que se encuentran a continuación de cada pregunta. Conteste cero (“0”) si no ha realizado dicha actividad en los últimos 3 meses. (Por favor, consulte el siguiente calendario si es necesario).

1. En los últimos 3 meses, ¿Cuántos días ha faltado al trabajo o a su centro educativo a causa de su dolor de cabeza?
2. En los últimos 3 meses, ¿Cuántos días se ha reducido su rendimiento a la mitad o más, en el trabajo o en el centro educativo a causa de su dolor de cabeza? (No cuente los días tenidos en cuenta en la pregunta 1, cuando no asistió al trabajo o al centro educativo).
3. En los últimos 3 meses, ¿Cuántos días no realizó las tareas domésticas a causa de su dolor de cabeza?
4. En los últimos 3 meses, ¿Cuántos días se ha reducido su rendimiento a la mitad o más, para realizar las tareas de casa a causa de su dolor de cabeza? (No cuente los días tenidos en cuenta en la pregunta 3, cuando no realizó las tareas domésticas).
5. En los últimos 3 meses, ¿Cuántos días dejó de asistir a actividades familiares, sociales o lúdicas a causa de su dolor de cabeza?
6. En los últimos 3 meses, ¿Cuántos días ha sufrido algún tipo de dolor de cabeza? (Si el dolor de cabeza duró más de un día cuente los días por separado).
7. En una escala de “0” a “10”, ¿Cuál fue la intensidad media de sus dolores de cabeza?
